# Shikonin Induces Autophagy and Apoptosis in Esophageal Cancer EC9706 Cells by Regulating the AMPK/mTOR/ULK Axis

**DOI:** 10.1155/2024/7752299

**Published:** 2024-10-29

**Authors:** Junli Zhang, Jiayi Guo, Biao Gu, Fen Wang, Yi Li, Ling Shang, Wendi Jiang, Junrao Ma, Wenjuan Wu

**Affiliations:** ^1^Bengbu Third People's Hospital Affiliated to Bengbu Medical University, Bengbu 233030, Anhui, China; ^2^Bengbu Medical University Key Laboratory of Cancer Research and Clinical Laboratory Diagnosis, Bengbu Medical University, Bengbu 233030, Anhui, China; ^3^Department of Biochemistry and Molecular Biology, School of Laboratory Medicine, Bengbu Medical University, Bengbu 233030, Anhui, China

**Keywords:** apoptosis, autophagy, esophageal cancer, shikonin, the AMPK/mTOR/ULK axis

## Abstract

Shikonin is a plant medicine extracted from *Lithospermum*, which dominate influential antioxidant and antitumor effect. Here, we report that shikonin was capable of inducing human esophageal cancer EC9706 cell apoptosis and autophagy, in a time- and dose-dependent manner. Shikonin exposure repressed cell viability and migration and invasion capabilities and caused EC9706 cell autophagy and apoptosis by activating the AMPK/mTOR/ULK axis. Autophagy inhibition secured EC9706 cells against shikonin-induced autophagy and apoptosis and reversed the upregulation of AMPK and ULK phosphorylation and downregulation of mTOR phosphorylation provoked by shikonin. In summary, shikonin instigates EC9706 cell apoptosis and autophagy using the target AMPK/mTOR/ULK signal pathway axis, which provides a potential new target to treat human esophageal cancer.

## 1. Introduction

Esophageal squamous cell carcinoma (ESCC) is one of the deadly tumors amidst malignant tumors in the digestive tract, mainly presenting with dysphagia and pain. Despite the continuous advancement of diagnostic and treatment techniques, its prognosis is still worrying [1, 2]. In 2020, the incidence rate ranked seventh, with 47,604 million new cases and with 544,076 deaths. China is in the group of the prone regions of ESCC across the globe [3]. Chemotherapy, surgery, and radiotherapy are the most frequent strategies to treat esophageal cancer [4, 5]. Chemotherapy is effective for late-stage ESCC (metastatic or dispersive) and recurrent ESCC [6]. Numerous drugs exist to treat ESCC in the clinical setting, such as cisplatin and 5-fluorouracil (5-FU) [7]. As it often causes gastrointestinal adverse effects similar to nausea and vomiting, some patients cannot tolerate it, and one can hardly achieve the expected result [8]. Moreover, the handicaps of 5-FU depend on imports and are not easily obtainable [9]. Consequently, it is compelling to discover a new targeted drugs for the treatment of ESCC.

The traditional Chinese medicine *Lithospermum* has a long history of medicinal use in China [10, 11]. Lithospermum is the dry root of the perennial herbs of the Boraginaceae family [12, 13]. It treats wet macula, purpura, hematuria, burn, eczema, erysipelas, and other diseases in the Chinese medicine clinic [14–16]. In recent years, with the continuous deepening of the research on Boraginaceae plants, it has been confirmed that the main practical components of Boraginaceae plants are a class of fat-soluble naphthoquinone pigments [17, 18]. Shikonin and its derivatives have multiple functions in terms of medicinal value, such as anti-inflammatory, antifertility, antitumor, bactericidal, and antivirus, liver protection, and anti-immunodeficiency effects [19]. Shikonin's structure was similar to that of daunorubicin and doxorubicin and had vigorous antitumor activity [20]. Liu et al. [21] announced that shikonin-inspired apoptosis and autophagy repressed the proliferation of human melanoma cells. Shikonin significantly triggered endoplasmic reticulum stress, intervened in cell apoptosis, and prompted protective autophagy via triggering the p38 pathway. After autophagy inhibitor inhibited autophagy of A375 cells, shikonin-inspired apoptosis was strengthened [21].

In triple-negative breast cancer, shikonin also significantly induced necrosis and apoptosis in a concentration- and time-dependent manner. It facilitates the autoubiquitination and degradation of apoptosis inhibitor protein, thus inhibiting the awakening of survival-promoting signaling pathways and accelerating cell necrosis [22]. Based on the above findings, shikonin inhibits the development of tumor cells in different ways. Nevertheless, the specific action mechanism of shikonin in esophageal cancer is not apparent. Zhang et al. [23] found that shikonin, a small-molecule inhibitor of pyruvate kinase 2 (PKM2), can impede glucose consumption, lactate production, and the process of glycolysis and pyruvate production. In addition, animal experiments also demonstrate similarity results [23]. However, the specific effects of shikonin on autophagy and apoptosis in ESCC have not been reported. Therefore, it is necessary to explore further how shikonin affects autophagy and apoptosis of ESCC to provide practical help for further research on the molecular mechanism of this compound and its clinical application.

In the current survey, the significant antitumor effect of shikonin in ESCC has been confirmed in vitro. The results revealed that shikonin notably inhibits malignant biological behavior of tumor cells, for instance, proliferation, migration, and invasion. Shikonin compels autophagy and apoptosis in EC9706 cells by regulating the AMPK/mTOR/ULK axis. As a result, it is speculated that shikonin can possibly be a new therapeutic strategy for ESCC.

## 2. Materials and Methods

### 2.1. Reagents and Antibodies

Shikimin, cisplatin, dorsomorphin (ComC), rapamycin (Rap), and Z-VAD-FMK were acquired from MedChemExpress (MCE, USA). Ampk (1:3000), phospho-Ampk (1:500), mTOR (1:1000), β-actin (1:5000), ULK (1:1000), phospho-ULK (1:1000), caspase3 (1:1000), cleaved caspase3 (1:1000), PARP (1:2000), cleaved PARP (1:2000), Bax (1:2000), and Bcl-2 (1:1000) all came from ProteinTech Group (Chicago, USA). Antibodies for LC3-Ⅰ/Ⅱ (1:2000), caspase8 (1:1000), cleaved caspase8 (1:1000), and phospho-mTOR (1:1000) were purchased from Cell Signaling Technology (Boston, USA).

### 2.2. Cell Lines and Cell Culture

The human ESCC9706 cells lines were purchased from the Chinese Academy of Sciences (Shanghai, China). Cells were cultured in RPMI‑1640 medium (Gibco, USA) with 10% fetal bovine serum (FBS) at 37°C.

### 2.3. Cell Proliferation Assay

The EC9706 cells were seeded into a 96-well plate in a 5% carbon dioxide (CO_2_) incubator for 24 h and then the shikonin and cisplatin (0, 0.25, 0.5, 1, 2, and 4 μM) for 24 and 48 h. Then, the Cell Counting Kit (CCK)-8 reagent (10 μL) (Beyotime, China) dissolved was added to each well and continuously cultured for 3 h in 5% CO_2_ (Thermo Scientific). The consequence was calculated at 450 nm. A statistic calculates shikonins half inhibitory concentration (IC50 value) on cell proliferation and, in addition, EC9706 cells with shikonin (0, 1 μM, and 4 μM) for 24, 48, 72, and 96 h. The cell proliferation activity was detected in the same way as above.

### 2.4. Transwell Assay

Cell suspension (2 × 10^5^ cells/200 μL) with 200 μL serum-free RPMI 1640 medium was added into the upper chamber, and then a total of 800 μL of medium containing 20% FBS was added into the lower chamber. After pretreatment with shikonin of different concentrations (0, 1 μM, and 4 μM) for 48 h at 37°C, the migration and invasion of cells are fixed and stained with crystal violet. Finally, the consequences were obtained by taking photos under a light microscope (Olympus).

### 2.5. Colony Formation Assay

A total of 8 × 10^4^ EC9706 cells were cultured on 6-well plates for 24 h and shikonin at different concentrations (0, 1 μM, and 4 μM). After 10–12 days, when the cell count is more than 50, the colonies are washed once with 1× phosphate-buffered saline (PBS) solution and then 4% paraformaldehyde for 15 min, and crystal violet is used for staining for 30 min. The colonies are imaged with a high-resolution camera.

### 2.6. Western Blotting Analysis

Collect cells, lyse them, and detect protein concentration. Adjust the protein concentration of each sample to 2 ug/mL, and use 12% sodium dodecyl sulfate PAGE to separate the protein. When the protein is transferred to the polyvinylidene fluoride (PVDF) membrane, seal the PVDF membrane with protein sealing solution at room temperature for 1 h and then incubate it in the first-antibody diluent at 4°C overnight. Then incubate the secondary antibody at room temperature. The density of the blot was conducted X-ray development and analysis.

### 2.7. Annexin V/Propidium Iodide (PI) Double Staining Assay

After the cells were treated for 48 h, and they were double-stained with fluorescein isothiocyanate (FITC)/PI apoptosis kit (Biotech Company) in accordance with the instructions. The cells were added with PI (8 μL) and annexin V-FITC and incubated for 15 min. The cell samples were analyzed by flow cytometry and FlowJo software.

### 2.8. Immunofluorescence Analysis

The cells inoculated on the tiny glass were exposed to 100% cold methanol for 15 min. After it permeabilized and blocked, subsequently incubate with primary LC3 antibody at 4°C overnight. The coverslips were incubated with secondary antibody IgG-AF488 (Life Technologies, USA) in the dark at 4°C for 1 h. Finally, the cells were treated with 4′,6-diamidino-2-phenylindole (DAPI) for 5 min, and the positive fluorescence signal of the green fluorescent protein spot (green) was detected under Zeiss LSM510 laser confocal microscope, and then analyzed LC3 signal with ImageJ software.

### 2.9. Statistical Analysis

All experimental data were analyzed using Statistical Package for the Social Sciences (SPSS) 25 and analyzed using GraphPad Prism 8.0 to generate statistical graphs. The experimental results were presented as the mean ± SD, and the experiment was independently repeated three times. Statistical differences for the two groups were performed with Student's *t*-test. For the comparison of three or more groups, one-way analysis of variance (ANOVA) was used when there was one variable, and two-way ANOVA of followed by a post hoc Tukey test was used when there were two variables. The level of statistical significance was set at ⁣^*∗*^*P* < 0 : 05, ⁣^*∗∗*^*P* < 0 : 01, and ⁣^*∗∗∗*^*P* < 0 : 001.

## 3. Results

### 3.1. Shikonin Inhibits EC9706 Cell Proliferation in a Dose- and Time-Dependent Manner

**Figure 1:**
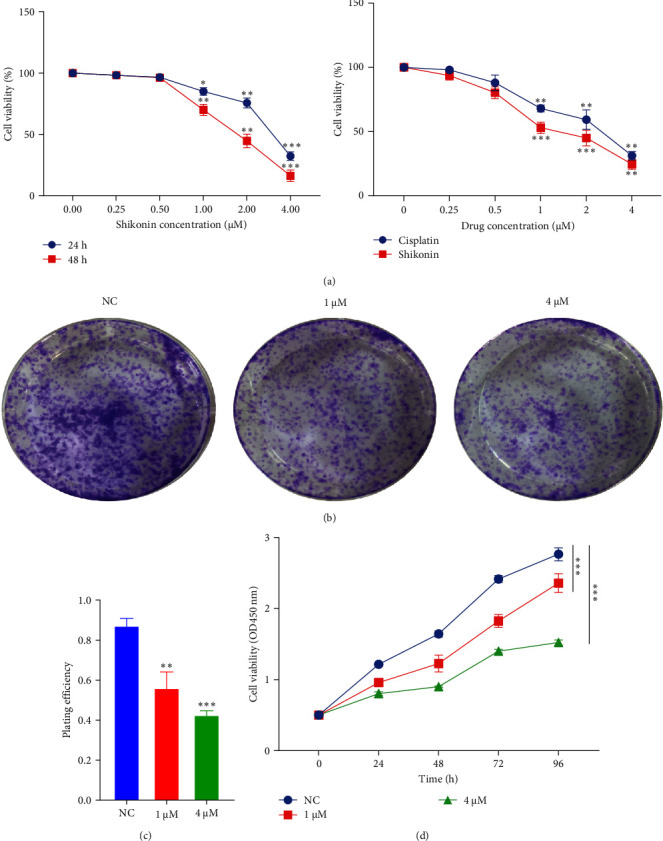
The effects of shikonin on EC9706 cell proliferation. (A) The cells were incubated with various concentrations of shikonin and cisplatin (0.25, 0.5, 1, 2, and 4 µM) for 24 and 48 h, and cell viability was measured by Cell Counting Kit (CCK)-8 assay. (B) Colony formation assay was employed to evaluate the proliferation of EC9706 cells. (C) The histogram represents the number of repeated bacteriolysis in each group based on colony formation measurements. (D) After 1 or 4 μM shikonin-treated EC9706 cells for 24, 48, 72, and 96 h, the CCK8 assay detected the cell proliferation. Data are represented as mean (*n* = 3) and standard deviation (SD) (⁣^*∗*^*P* < 0.05, ⁣^*∗∗*^*P* < 0.01, and ⁣^*∗∗∗*^*P* < 0.001).

Shikonin, a famous traditional Chinese medicine, has chemopreventive and therapeutic properties against a variety of tumors. To determine whether shikonin inhibits the proliferation of EC9706 cells, after treating cells with different concentrations of shikonin, the CCK8 assay can detect changes in cell viability. The consequences proved that the shikonin group (0.25, 0.5, 1, 2, and 4 µM) had a significant decrease in cell activity, as against the normal control (NC) group. Meanwhile, the EC9706 cell proliferation activity progressively lowered with the intensification of drug concentration (Figure 1A). The average cell survival rates at 48 h were 98.33 ± 0.94%, 96.33 ± 0.47%, 71.33 ± 2.63%, 44.67 ± 2.05%, and 17.67 ± 2.05%, respectively. Additionally, a control drug group was used to demonstrate the unique inhibitory effect of shikonin on cell proliferation. Cisplatin is a classic chemotherapy drug used to treat esophageal cancer. The experiment found that after 48 h of treatment with shikonin and cisplatin (0.25, 0.5, 1, 2, and 4 µM), the cell viability of both the shikonin group and cisplatin group decreased significantly with increasing drug concentration, and the decrease in cell viability was more pronounced in the shikonin group. Compared to cisplatin, shikonin has a better inhibitory effect on the proliferation of esophageal cancer cells (Figure 1A). After 48 h of treatment, the IC50 of shikonin on EC9706 cells was 2.26 ± 0.12 µM. In the following experiment, we chose the concentration of 1 and 4 µM to treat EC9706 cell. Furthermore, we also examined whether shikonin's inhibition of cell viability is time-dependent. At the concentrations of 1 and 4 µM, shikonin showed evident inhibition effects on EC9706 cell viability at 24 and 48 h, and shikonin treatment of cells at 48 h had a more substantial inhibitory effect on cell viability than at 24 h (Figure 1D). Similarly, the colony formation assay also showed similar results. After 48 h of treatment with shikonin, the number of colonies considerably declined (Figure 1B,C). These findings denote that shikonin restrains ESCC cell proliferation in a dose- and time-dependent manner.

### 3.2. Shikonin Repressed the Migratory and Invasive Abilities of EC9706 Cells

**Figure 2:**
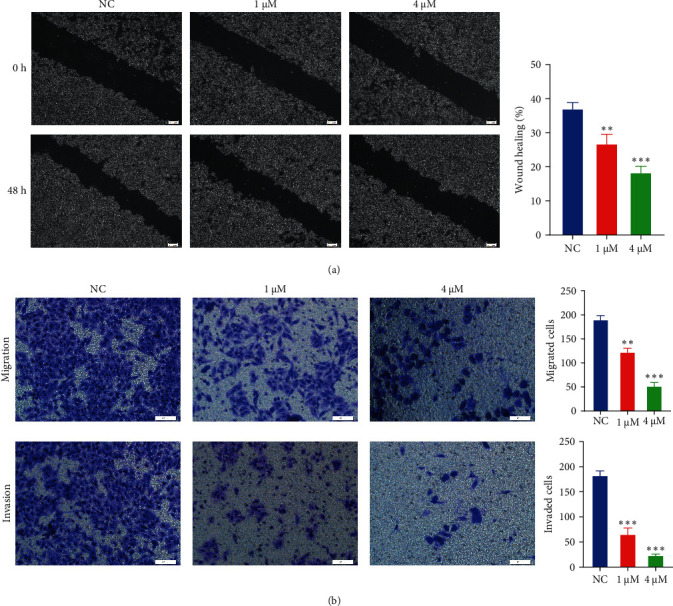
The inhibitory effect of different concentrations of shikonin on migratory and invasive abilities of EC9706 cells. (A) The size of the scratches measured by inverted fluorescence microscopy at 0 and 48 h in the three different groups of EC9706 cells. The images were captured at 40×. Histograms provide quantitative data on wound healing. (B) The Transwell assay detected the migration and invasion of EC9706 cells, which penetrate the polycarbonate membrane in the lower chamber that are stained purple. Compared with the normal control (NC) group, ⁣^*∗∗*^*P* < 0.01 and ⁣^*∗∗∗*^*P* < 0.001.

In the EC9706 cell line, the results found that the migration ability of cells was significantly different in shikonin groups in contrast with NC group. The wound healing of the shikonin group cells was delayed, and cell wound healing rate were 26.50% and 18.08%, respectively (Figure 2A). Shikonin substantially repressed the migration of EC9706 cell. Subsequently, the Transwell assay was used to observe the effect of shikonin on cell migration and invasion ability, and the outcome revealed that shikonin meaningfully reduced the number of cells migrating and invading the inferior cavity, and the migratory and invasive capacity of EC9706 cells diminished by 50.01% and 21.70%, respectively (Figure 2B).

### 3.3. Shikonin Induces Apoptosis in EC9706 Cells

**Figure 3:**
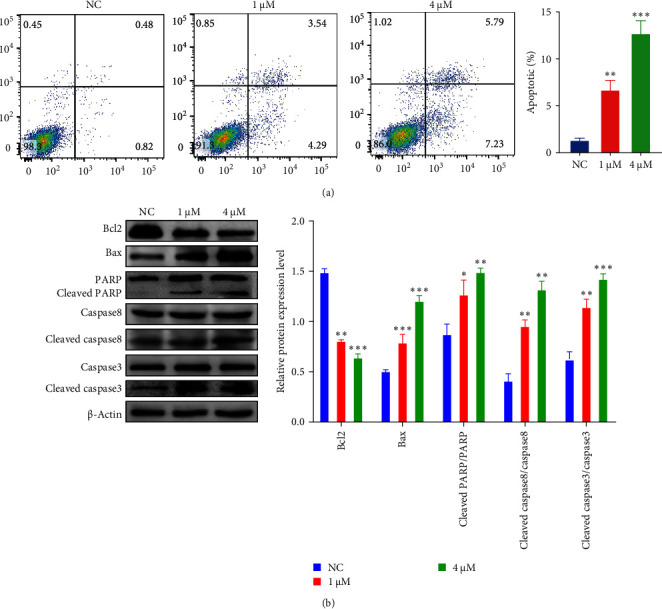
The inhibitory effect of shikonin on EC9706 cell apoptosis. (A) Apoptosis was detected by FITC/PI double staining. The results are expressed as a percentage of the normal control (NC), which is set to 100%. (B) After being treated with different concentrations of shikonin for 48 h, apoptosis-related (RAPA, caspase3, caspase8, Bax, and Bcl2) protein expression was analyzed by western blotting. The data are expressed as the mean ± standard deviation of three independent experiments. ⁣^*∗*^*P* < 0.05, ⁣^*∗∗*^*P* < 0.01, and ⁣^*∗∗∗*^*P* < 0.001.

In order to further investigate whether shikonin inhibits EC9706 cell growth by inducing apoptosis, we evaluated the apoptotic death of EC9706 cell after diverse doses of shikonin for 48 h. Apoptosis cell number was analyzed by annexin V/PI flow cytometry. Shikonin exposure induced EC9706 cell apoptosis (Figure 3A). The result shows that 1 and 4 µM shikonin increased the apoptosis percentage from 1.3% in the NC group to 7.83% and 13.02%, respectively (Figure 3A). The early apoptosis percentage was raised from 0.48% to 5.79%, and the late apoptosis percentage arose from 0.82% to 7.23% in EC9706 cells (Figure 3A). Moreover, the western blotting exhibited that only Bcl-2 protein expressions were decreased; nevertheless, the protein expression of Bax, cleaved caspase3, cleaved caspase-8, and cleaved PARP were elevated with increasing concentrations of shikonin (Figure 3B). Collectively, shikonin can induce EC9706 cell apoptosis in a dose-dependent. The above outcomes demonstrated that apoptosis present an essential role in the inhibition of EC9706 cell growth induced by shikonin.

### 3.4. Shikonin Induces Autophagy in EC9706 Cells

**Figure 4:**
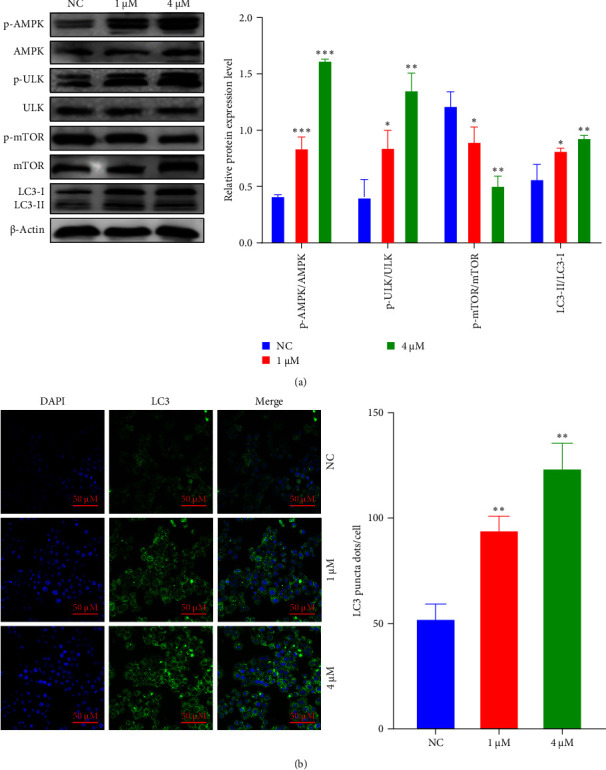
The inhibitory effect of shikonin on EC9706 cell autophagy. (A) Western blot analysis was used to determine the protein (p-AMPK, p-ULK, p-mTOR, and LC3) levels in EC9706 cells. ⁣^*∗*^*P* < 0.05, ⁣^*∗∗*^*P* < 0.01, and ⁣^*∗∗∗*^*P* < 0.001 versus the normal control (NC) (*n* = 3). (B) The LC3 puncta were examined using confocal microscopy and were quantified. LC3 is shown in green. 4′,6-diamidino-2-phenylindole (DAPI) is shown in blue, which stained the nuclei. Confocal microscope was taken at ×20.

To appraise the essential function of shikonin in the autophagy of ESCC cells, this study analyzes the expression of autophagy essential protein LC3 in ESCC cells. In the western blotting assay, shikonin treatment of EC9706 cells led to an increase in LC3- II protein expression intracellularly, pointing that shikonin may be an essential part in the autophagic of ESCC (Figure 4A). To visually present the LC3 protein distribution, an immunofluorescence assay was performed, which was consistent with western blotting results. Research determines that after the treatment with shikonin, the LC3 protein content of EC9706 cells was greatly increased in contrast with the NC group (Figure 4B). In conclusion, our results indicate that shikonin induces autophagy in EC9706 cells. To continue studying the potential mechanism of shikonin instigating autophagy, the research found that shikonin treatment significantly promoted phosphorylation levels of p-AMPK and p-ULK and diminished p-mTOR. In contrast, the total AMPK, ULK, and mTOR did not alter considerably. The above consequences indicate that shikonin can induce autophagy of EC9706 cells.

### 3.5. Shikonin-Induced Autophagy and Apoptosis Through Activation of AMPK/mTOR/ULK Pathway

**Figure 5:**
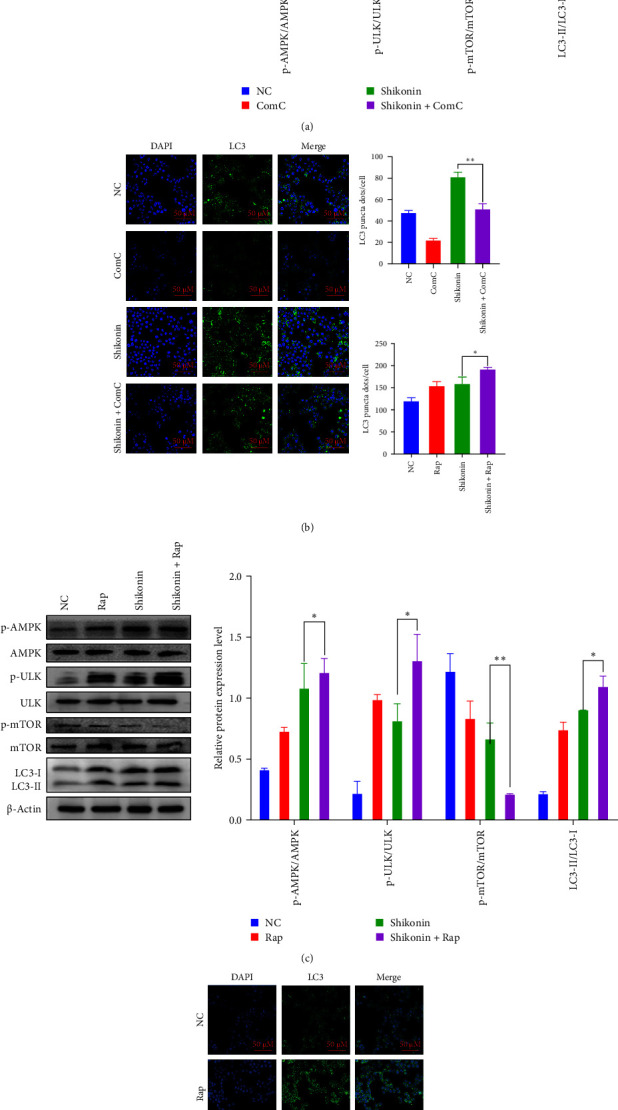
Shikonin-induced autophagy and apoptosis through activation of AMPK/mTOR/ULK pathway. (A and C) After exposed to ComC or Rap for 24 h, followed by adding shikonin to treat the cells for 24 h, the expression of autophagy and pathway-related proteins was detected by western blotting, with β-actin, which was used as a loading normal control (NC). (B and D) After 24 h of Rap or ComC pretreatment, the cells were exposed to shikonin for 24 h, and immunofluorescence assay (×20) detected LC3 puncta of EC9706 cells. Data are presented as the mean ± standard deviatio (SD) of three independent experiments (*n* = 3, ⁣^*∗*^*P* < 0.05, ⁣^*∗∗*^*P* < 0.01, and ⁣^*∗∗∗*^*P* < 0.001) for shikonin group versus shikonin+ Rap or shikonin+ ComC (group).

**Figure 6:**
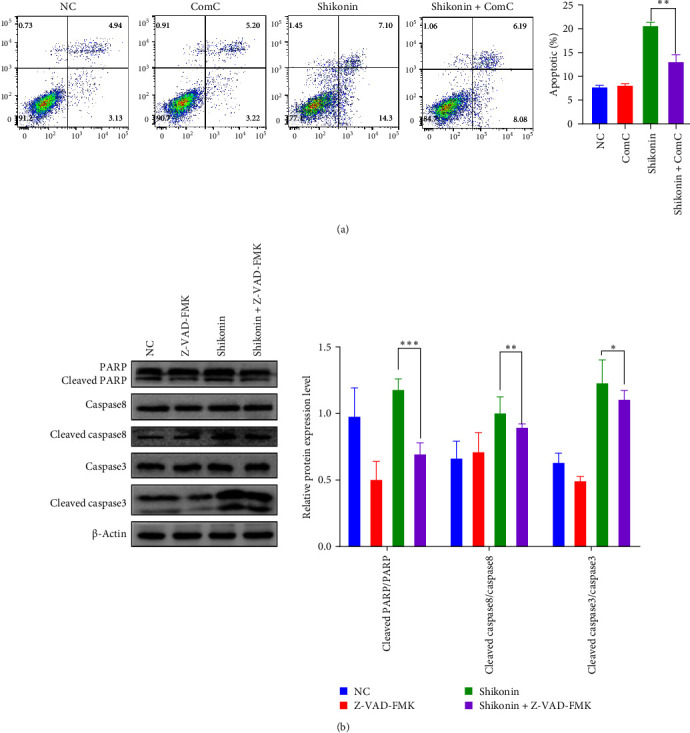
The combination of shikonin and ComC or the apoptosis inhibitor Z-VAD-FMK (ZVF)-induced cell apoptosis. (A) Apoptosis in EC9706 cells treated for 48 h as shikonin alone or in combination with ComC or the apoptosis inhibitor ZVF was examined by flow cytometry. (B) Western blotting analysis was used to determine the apoptosis protein levels in EC9706 cells. ⁣^*∗*^*P* < 0.05, ⁣^*∗∗*^*P* < 0.01, and ⁣^*∗∗∗*^*P* < 0.001 versus the normal control (NC) (*n* = 3).

To confirm that shikonin instigates autophagy by AMPK/mTOR/ULK axis, we treated cells with Rap (an mTOR inhibitor) and ComC (an inhibitor of AMPK, autophagy inhibitor). Firstly, the cells are exposed to ComC for 24 h, followed by adding shikonin to treat the cells for 24 h. AMPK, ULK, and mTOR activation were examined using a western blotting assay employing phosphorylated antibodies. Pretreatment significantly inhibited shikonin-induced p-AMPK and p-ULK, increasing p-mTOR and reducing it. Compared with shikonin treatment alone, ComC and shikonin significantly reduced the accumulation of LC3-II (Figure 5A). Besides, we performed immunofluorescence technology to determine the effects of the combination treatment with ComC and shikonin, and results illustrated that the proportion of LC3-positive autophagosomes significantly reduced. ComC significantly weakened the promotion of shikonin on autophagy of EC9706 cells (Figure 5B). Moreover, compared with shikonin alone, shikonin combined with ComC can dramatically reduce the number of apoptosis, and ComC can reduce the apoptosis induced by shikonin (Figure 6A). To ascertain whether mTOR engages in shikonin-induced autophagy, after 24 h of Rap pretreatment, the cells were exposed to shikonin for 24 h. EC9706 cells pretreated with shikonin and Rap significantly increased p-AMPK and p-ULK and diminished p-mTOR. Compared with shikonin alone, Rap combined with shikonin increased the accumulation of LC3-II (Figure 5C). Similarly, Rap significantly increased the percentage of cells expressing LC3-positive autophagosomes induced by shikonin (Figure 5D). In addition, Z-VAD-FMK (ZVF), an apoptosis inhibitor, can verify the EC9706 cell apoptosis prompted by shikonin. After 48 h of combined treatment with shikonin and ZVF on cells, the outcomes showed that ZVF combined with shikonin pretreatment appreciably lowered the protein levels of cleaved caspase3, cleaved PARP, and cleaved caspase8 and significantly saved the apoptosis induced by shikonin (Figure 6B). In summary, these data indicate that shikonin compels autophagy and apoptosis via triggering AMPK/mTOR/ULK pathway.

## 4. Discussion

In recent decades, the incidence rate of patients with ESCC has enhanced significantly, and the trend is increasing year by year [9, 24]. They characterized that they have poor response to chemotherapy, high invasion, and metastasis potential [1]. The prognosis of ESCC is that it is difficult to achieve the expected results; as less than half of the patients are eligible for surgical treatment, percutaneous ablation, and other treatments, most patients do not have good treatment options [4, 25]. Therefore, there is a need to actively search for molecular targets of ESCC and provide targeted treatment for it [26]. The main effective ingredient of traditional Chinese medicine Lithospermum is shikonin [27, 28]. The researches have displayed that shikonin can exert antitumor effects through various pathways, such as causing cell apoptosis and suppressing cell growth and antiangiogenesis [22, 29]. Various tumor in vivo experiments in xenografted, allografted, and orthotopic tumor models, such as colorectal cancer [30], colon cancer [31], and human non–small cell lung cancer [32], have revealed that shikonin initiates tumor cell-killing activity by multiple mechanisms, including the stimulation of apoptosis, autophagy, and necroptosis and the inhibition of angiogenesis.

A clinical trial report stated that using shikonin to treat advanced lung cancer patients inhibited tumor growth and improved their immune function. The tumor diameter is reduced by more than 25%, the response rate is 37%, and the 1-year survival rate is 47%. The quality of life of patients has also been significantly improved, and shikonin has no adverse effects on the liver, heart, kidneys, and blood [33]. Another study used the keywords “purple grass extract” or “purple grass root” in ClinicalTrails.gov (https://clinicaltrials.gov/); on the website search, it was found that there are currently five clinical trials related to shikonin (as of September 10, 2021). Although there are few clinical registration studies related to shikonin, shikonin and its derivatives have been shown to inhibit the growth of various types of tumors in vitro and in vivo. Preliminary clinical trials also prove that translating these effects into clinical practice is possible [34].

This research mainly focuses on examination whether shikonin can be used as an anticancer drug for the treatment of ESCC. Our evidences revealed that shikonin impedes EC9706 cell growth and reduces the ability of EC9706 cells to migrate and invade. Meantime, shikonin restrains the growth of EC9706 cells by way of regulating changes between autophagy and apoptosis. Shikonin performances appear as upregulating proapoptotic protein caspase- and cleaved PARP, increasing Bax/Bcl-2 ratio, indicating EC9706 cell apoptosis, accompanied by improved LC3 levels and accelerated p62 degradation, reflecting induced EC9706 cell autophagy. This experiment reversed the autophagy and apoptosis of EC9706 cells induced by shikonin by using autophagy and apoptosis inhibitors, and autophagy activators can enhance the activation of shikonin.

Autophagy inhibits the occurrence and development of cancer and is expected to become a molecular target for treating cancer [35]. The relationship between autophagy and apoptosis is very complex, involving molecules that interact, coordinate, and antagonize each other, inducing tumor cell death and growth in different ways [36]. For instance, in human bladder cancer cells, using autophagy pathway activators and inhibitors, it was found that artesunate-inducing cells cause autophagy-dependent apoptosis and upregulation of reactive oxygen species (ROS) which cause this effect [37]. Research using Rap and 3-MA found that antioxidant berberine can reduce ROS production, suppress autophagy and apoptosis in cells, and promote cell growth [38]. In prediction models for autophagy-related genes, multiple autophagy genes are involved, such as ATG10, ATIC, BIRC5, and CAPN10, and their changes effectively affect the survival time of hepatocellular carcinoma patients [39].

The activation of autophagic or enhancement of autophagic cell activity is involved in the progression of ESCC [8]. In EC9706 cells, we found that shikonin-induced LC3 fluorescence spots increased, which gradually increase LC3-II and decrease LC3-I. Similarly, shikonin can upregulate the protein expression of LC3 in EC9706 cells and increase the proportion of LC3-I/LC3-II. Rap can significantly activate autophagy by inhibiting mTOR way, and it can promote autophagy of EC9706 cells induced by shikonin. ComC is an autophagy inhibitor that inhibit the formation of autophagosomes [40]. In EC9706 cells, ComC inhibits autophagy, in which the conversion from LC3-I to LC3-II and the increased expression of LC3 induced by shikonin are restrained, and the collection of p62 is raised. The death of ESCC cells depends on the awakening of caspase3 due to shikonin, showing that dysfunctional autophagy contributes to the caspase3-dependent apoptosis triggered by shikonin. The drug inhibition test proved that the combination treatment with ComC can significantly prevent the activation of caspase3 triggered by shikonin and partially protect cells from the toxic effects of shikonin. At the same time, incubation with ZVAD-FMK, a pan-caspase inhibitor, blocked ESCC cell death mediated by shikonin. Consequently, our findings indicate that there is a robust interaction association between autophagy and apoptosis.

Recently, research also shows that *Salvia chinensia* Benth treat esophageal cancer cells which resulted in the upregulation of p-AMPK and p-ULK1 expression, activation of signaling pathways, and increased accumulation of autophagy marker LC3 [41]. However, when studying the effect of camptothecin on cell autophagy, it was found that the AMPK/mTOR/ULK1 axis plays a protective role in autophagy in ESCC [42]. This suggests that autophagy regulation may be an attractive strategy. The AMPK and mTOR can regulate autophagy by directly phosphorylating ULK1 and ULK complexes [43]. The AMPK/mTOR signaling pathway is the primary adjuster of autophagy [44]. Thus, we studied how the AMPK/mTOR/ULK pathway affects autophagy and apoptosis. It is well-known that mTOR is an autophagic inhibitor that binds ULK and maintains phosphorylation [45]. The activity of mTOR complex helps cells cope with different stimuli, thereby regulating cell growth and cell death processes [46]. Inhibiting mTOR kinase activity activates downstream substrate ULK while also inhibiting downstream important regulatory protein Bcl-1, promoting cell autophagy [47]. To further distinguish the function of mTOR in shikonin-triggered autophagy, shikonin and autophagy activator Rap were jointly treated, which resulted in a tremendous enhancement in shikonin which caused LC3-II level in an EC9706 cell line. Besides, coincubation of cells with ComC decreased LC3-II, AMPK, and ULK-phosphorylated levels, further saved mTOR phosphorylation, and decreased shikonin-induced apoptosis. Therefore, we suggest that shikonin-induced autophagic cell death may depend on adjusting the AMPK/mTOR/ULK axis and then activating autophagy-dependent apoptosis. We speculate that shikonin may first inspire the phosphorylation of AMPK, inhibiting the mTOR complex and thus activating the downstream substrate ULK. These results further indicate that shikonin may participate in autophagy and apoptosis of EC9706 cells via the AMPK/mTOR/ULK axis. There is still a lack of research on the side effects of shikonin, and shikonin lacks specific selectivity for cancer cells, which can lead to normal cell toxicity [48]. Shikonin derivatives have selective toxicity toward MDA-MB-231 and MCF-7 cancer cells and are not toxic to normal cells [49]. Another study found that shikonin compound exhibits mild toxicity to normal human skin fibroblasts [50]. In addition, more clinical research is needed on the synergistic treatment of shikonin with other anticancer drugs [34]. Further research should be conducted to address these issues in order to widely apply shikonin in antitumor clinical treatment as soon as possible.

## 5. Conclusions

As a conclusion, this research revealed that shikonin inhibits EC9706 cell proliferation by adjusting the AMPK/mTOR/ULK signal pathway for the first time, thereby stimulating autophagic apoptosis. These data reveal the antitumor mechanism of shikonin, which helps establish shikonin as a new therapy for ESCC.

## Data Availability

The datasets used and/or analyzed during the current study are available from the corresponding author on reasonable request.
